# AVNRT complicated by Valsalva-exacerbated radial pulsation artifacts mimicking acute ischemia: a case report

**DOI:** 10.3389/fcvm.2025.1612805

**Published:** 2025-06-19

**Authors:** Peng Li, Jingyu Kan, Jingwen Ding, Chongkai Liu, Huaisheng Ding

**Affiliations:** ^1^ Department of Cardiology, Meishan People’s Hospital, Meishan, Sichuan, China; ^2^ Department of Internal Medicine, Pengshan District Traditional Chinese Medicine Hospital, Meishan, Sichuan, China

**Keywords:** case report, AVNRT, ECG artifact, ST-T changes, Valsalva maneuver

## Abstract

Atrioventricular nodal reentrant tachycardia (AVNRT), the most common supraventricular tachycardia, occasionally present with transient ST-T changes mimicking ischemia, posing diagnostic challenges for patients. We report an 82-year-old woman with paroxysmal palpitations whose initial ECG demonstrated supraventricular tachycardia (154 bpm) accompanied by dynamic ST-segment elevation and T-wave inversion in leads I, II, aVL, and aVF, while lead III remained unaffected. Repositioning electrodes away from the right radial artery pulsation resolved these deviations, confirming their artifactual origin. The electrophysiological study confirmed the diagnosis of atrioventricular nodal reentrant tachycardia (AVNRT), and successful radiofrequency catheter ablation was performed with no recurrence during 6-month follow-up. Notably, breath-holding during tachycardia exacerbated pulsation artifacts by altering hemodynamic forces, a phenomenon further validated through provocative Valsalva maneuvers. This case highlights that limb pulsation artifacts—localized via Einthoven's triangle principles (sparing lead III)—can mimic ischemic patterns, particularly under tachycardia and breath-holding. Clinicians should prioritize ECG artifact exclusion through lead adjustment and dynamic testing to avoid unnecessary invasive interventions.

## Introduction

Atrioventricular nodal reentrant tachycardia (AVNRT), the most prevalent form of paroxysmal supraventricular tachycardia (SVT), accounts for approximately 60% of narrow QRS complex tachycardias (1). While AVNRT predominantly affects women without structural heart disease, its clinical presentation—characterized by abrupt palpitations, dizziness, or chest discomfort—often overlaps with symptoms of acute coronary syndromes (2). Electrocardiographically, transient ST-segment depression or T-wave inversion (ST-T deviations) occurs in up to 25% of SVT cases, mimicking ischemic patterns and complicating diagnostic differentiation (3). These repolarization abnormalities are attributed to rate-related repolarization heterogeneity, autonomic dysregulation, or retrograde atrial conduction (4). However, physiological artifacts—such as limb arterial pulsation (e.g., radial artery) or somatic tremors—represent an underrecognized confounder in ECG interpretation, particularly during tachycardia (5).

In clinical practice, distinguishing between genuine ischemia and ECG artifacts requires careful consideration, given that misinterpretation might result in unnecessary examination that could otherwise be avoided. While prior studies have documented pulsation-related artifacts (6–8), few emphasize the dynamic interaction between tachycardia and physiological maneuvers (e.g., Valsalva) in amplifying artifact generation. Moreover, the role of Einthoven's triangle principles in localizing limb-derived artifacts—such as sparing a single lead (I, II, or III)—is seldom highlighted in clinical practice (5).

Herein, we report a case of AVNRT in which right radial artery pulsation artifacts—exacerbated by breath-holding during tachycardia—induced transient ST-segment elevation and T-wave inversion, mimicking ischemic patterns.

## Case report

**Figure 1:**
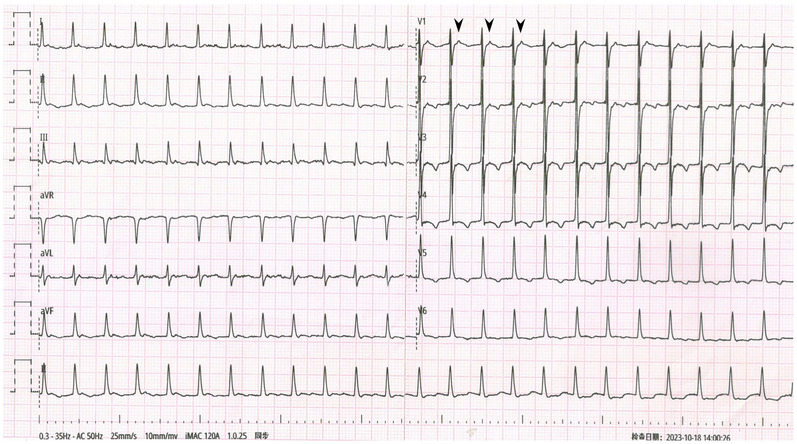
Admission ECG showing SVT with pseudo ST-segment elevation and pseudo T-wave inversion. Black arrowheads: P’wave. Red arrowheads: Pseudo ST-segment elevation and pseudo upright T-wave. Yellow arrowheads: T-wave transformed into pseudo bidirection. Blue arrowheads: T-wave transformed into pseudo inversion.

**Figure 2:**
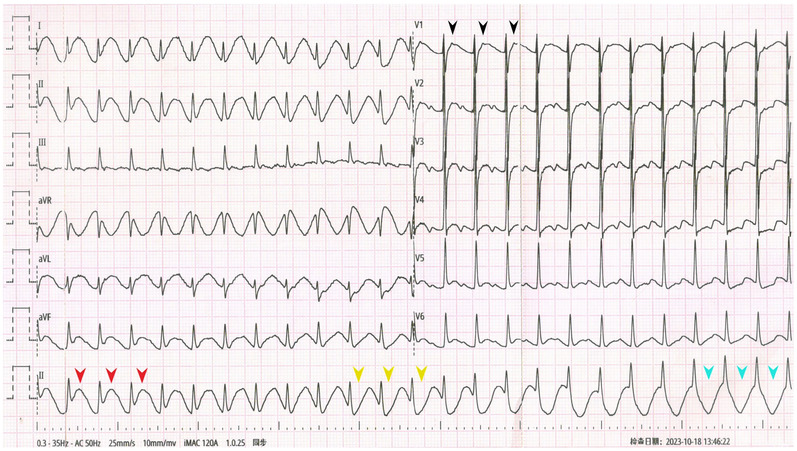
The repeat ECG showed SVT without obvious ST-T deviation.

An 82-year-old woman with a height of 1.65 m and a weight of 55 kg (BMI 20.20 kg/m^2^) came into our center for treatment of paroxysmal palpitations. She had no chest pain or chronic comorbidities and was not taking any medicine. No abnormalities were found in echocardiography and results of blood investigations were within normal limits. 12-lead electrocardiogram (ECG) was done using a digital multi-channel electrocardiograph (iMAC 100, Zoncare Biomedical Electronics Co., Shenzhen, China; filter setting: 0.05–150 Hz) when admitted (Figure 1). Initial evaluation noted ST-segment abnormalities prompting consideration of ischemic etiology. Given the absence of chest pain and normal serial cardiac biomarkers, the senior physician recommended a repeat ECG to investigate the discordance between electrical and clinical findings (Figure 2). The repeated 12-lead ECG was also using the same electrocardiogram instrument and parameters with the patient in supine position and standard limb lead placement (arms/legs away from major arteries).

The admission ECG showed supraventricular tachycardia (SVT) with heart rates of 154 beats per minute (bpm). QRS interval was 96 ms followed by P' wave (black arrowheads). In leads I, II, aVL and aVF, pseudo ST-segment elevation and upright T-wave were observed (red arrowheads) and gradually ST-segment turned pseudo depression and T-wave transformed into pseudo bidirection (yellow arrowheads) and pseudo inversion (blue arrowheads) in lead II. No obvious abnormality was observed only in lead III among limb leads. The repeated ECG showed SVT without obvious ST-T deviation.

**Figure 3:**
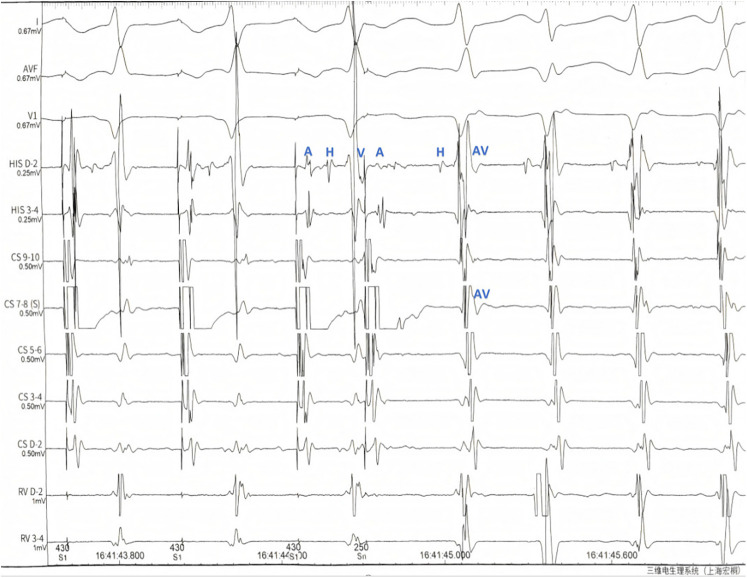
Electrophysiological Observations: During programmed stimulation (S1S2 430/280 ms): (1) Dual atrioventricular nodal physiology was demonstrated by: A significant AH interval prolongation (the AH interval prolonged by 52 ms compared to baseline). Clear AH “jump” phenomenon (>50 ms diagnostic threshold). (2) Tachycardia induction characteristics: Consistent with typical AVNRT pattern. Manifested by: Ventriculoatrial (VA) fusion on CS catheter2.HA interval <70 ms during tachycardia. Key notations: A, atrial potential; H, his bundle potential; V, ventricular potential; ms, milliseconds.

Comparing the two ECGs, the ST-T change in Figure 1 was considered to be caused by artifacts from right radial pulsation. Electrophysiological examination was done that day, AVNRT was confirmed (Figure 3) and and successfully treated with radiofrequency ablation. During the 6-month follow-up, the patient's arrhythmia did not recur.

## Discussion

AVNRT is the most common SVT (1). ST-segment depression, T-wave inversion, or both are observed in about 25% of AVNRT cases (2). Various mechanisms may account for the ST-segment depression seen during SVT, such as differences in action potentials in different regions during systole, retrograde atrial activation, or reflex neurogenic mechanisms (3). In our patient, the pseudo ST-T wave pattern was considered to be an artifact caused by the pulsation of the right radial artery, as the lead III was normal.

Sources of ECG artifacts can be divided into 2 categories generally: physiological and nonphysiological (4). Physiological sources may include somatic muscle tremor, excessive movement and arterial pulsation. Artifacts from pulsation have been reported before, such as: arm arteriovenous fistula (6), atypical radial artery (7) or abdominal aortic pulsation (8). According to the Einthoven triangle theory (5), lead I compares the electrical differences between the right arm and left arm; lead II, between the right arm and left leg; and lead III, between the left arm and left leg. Therefore, when the artifacts originate from the left arm, the lead between the right arm and left leg (lead II) remains normal. Similarly, when the artifacts originate from the right arm, the lead between the left arm and left leg (lead III) remains normal. And when the artifacts originate from the left leg, the lead between the left arm and right arm (lead I) remains normal. Moreover, limb artifacts can also affect precordial leads because the Wilson central terminal is produced by connecting 3 limb electrodes via resistive network to give an average potential across the body (8). Therefore, only one limb lead (I, II, or III) will remain unaffected when a single limb is the source artifact. This is an important and simple method for detecting the ECG artifacts caused by pulsation originating from a single limb.

In previous reports (5–7), artifacts caused by arterial pulsation had fixed interval with QRS complex, while in our case, the interval between the artifact and the QRS complex was not fixed and tended to gradually increase. Careful observation of the ST-T changes reveals the real course of T wave (artifacts) gradually approaching the next QRS complex, which had never been reported before. We thought the change of “T wave” might be caused by delayed cardiac pumping adapted from breath-hold. Then, the patient was told to perform Valsalva maneuver when recording one more ECG, the similar ST-T change was observed again.

The Valsalva Maneuver can lead to a decrease in cardiac output, especially when supine position. We considered that the progressive decrease in cardiac output leads to a gradual delay in pulse waves, resulting in this special artifacts (9). However, when the patient's heart rhythm returned to normal sinus rhythm (less than 100 bpm), even if the patient performed the Valsalva maneuver again, it couldn't trigger the aforementioned gradual pseudo ST-T changes.

Therefore, we speculated that the key mechanism underlying the observed pseudo ST-T changes lies in the interaction between tachycardia and hemodynamic alterations during breath-holding. As cardiac output decreased during the Valsalva maneuver, the radial artery pulsation artifact became temporally dissociated from the QRS complex. This dissociation resulted in a variable coupling interval, with the artifact progressively merging into the subsequent QRS complex. Consequently, the ECG displayed pseudo ST-segment elevation (artifact peak mimicking ST elevation) and pseudo T-wave inversion (artifact trough overlapping with the T-wave segment).

## Conclusion

This case highlights the critical importance of distinguishing true ischemic ST-T changes from physiological artifacts in patients with paroxysmal supraventricular tachycardia (SVT). While transient ST-segment deviations are observed in up to 25% of SVT cases, artifacts—particularly those caused by limb arterial pulsation—can mimic pathological patterns, leading to potential misdiagnosis. The unaffected lead III in this patient, consistent with Einthoven's triangle principles, and resolution of ST-T abnormalities upon electrode repositioning provided clear evidence of artifact origin (3). Clinicians should remain vigilant in asymptomatic patients, utilizing repeat ECGs with adjusted lead placement or provocative maneuvers (e.g., Valsalva) to unmask artifactual changes. Furthermore, this case underscores that tachycardia combined with breath-holding may exacerbate pulsation-related artifacts by altering hemodynamic forces, a phenomenon warranting further mechanistic investigation. Recognizing these nuances not only prevents unnecessary interventions but also refines diagnostic accuracy in SVT management. Future studies should explore standardized protocols for artifact differentiation and their integration into clinical guidelines.

## Data Availability

The original contributions presented in the study are included in the article/Supplementary Material, further inquiries can be directed to the corresponding author.

## References

[1] Page RL Joglar JA Caldwell MA Calkins H Conti JB Deal BJ 2015 ACC/AHA/HRS guideline for the management of adult patients with supraventricular tachycardia. Circulation. (2016) 133(14):26. 10.1161/CIR.0000000000000311 26399663

[2] Riva SI Della BP Fassini G Carbucicchio C Tondo C. Value of analysis of ST segment changes during tachycardia in determining type of narrow QRS complex tachycardia. J Am Coll Cardiol. (1996) 27(6):1480–5. 10.1016/0735-1097(96)00013-7 8626962

[3] Nelson SD Kou WH Annesley T de Buitleir M Morady F. Significance of ST segment depression during paroxysmal supraventricular tachycardia. J Am Coll Cardiol. (1988) 12(2):383–7. 10.1016/0735-1097(88)90410-x 3392331

[4] Zhai H Zheng B Zhai G. Is a shaking hand or a trembling heart producing changes in electrocardiogram findings? JAMA Intern Med. (2022) 182(7):772–3. 10.1001/jamainternmed.2022.1796 35604652

[5] Aslanger E Yalin K. Electromechanical association: a subtle electrocardiogram artifact. J Electrocardiol. (2012) 45(1):15–7. 10.1016/j.jelectrocard.2010.12.162 21353235

[6] Sotananusak T Meemook K. Asymptomatic ST-segment–elevation ECG in patient with kidney failure. Circulation. (2018) 137(4):402–4. 10.1161/CIRCULATIONAHA.117.032657 29358346

[7] Zhai HL He Y. Transient ST-segment elevation and QTc interval prolongation in a patient with persistent chest pain. JAMA Intern Med. (2021) 181(12):1652–3. 10.1001/jamainternmed.2021.6015 34724533

[8] Al Saiegh Y Liskov S Yan G. Spiked helmet sign in the Inferior leads. JAMA Intern Med. (2023) 183(9):1007–8. 10.1001/jamainternmed.2023.1728 37428496

[9] Mäntysaari M Antila K Peltonen T. Relationship between the changes in heart rate and cardiac output during the Valsalva maneuver. Acta Physiol Scand Suppl. (1984) 537:45–9. 6596862

